# Endoscopic submucosal excavation for a small rectal gastrointestinal stromal tumor with serosal layer preservation: a case report

**DOI:** 10.3389/fonc.2026.1738939

**Published:** 2026-03-12

**Authors:** Yu Zhou, Mei Yuan, Ge Yu, Jie Xu, Zhaoyi Chen, Daoxing He

**Affiliations:** 1 Department of Gastroenterology, Xuancheng People’s Hospital, Affiliated Xuancheng Hospital Wannan Medical College, Xuancheng, Anhui, China; 2 Department of Gastroenterology, Shanghai General Hospital, Shanghai Jiaotong University School of Medicine, Shanghai, China; 3 Electrocardiogram Room, Huai’an No.3 People’s Hospital, Huai’an, Jiangsu, China

**Keywords:** endoscopic submucosal excavation, endoscopic ultrasonography, gastrointestinal stromal tumor, muscularis propria, rectal gastrointestinal stromal tumor

## Abstract

**Background:**

Gastrointestinal stromal tumor (GIST) is the most common mesenchymal tumor of the gastrointestinal tract, with rectal involvement being relatively rare, accounting for approximately 5% of all GISTs. Due to the unique anatomy of the rectum, most rectal GISTs originate from the muscularis propria, which increases the risk of perforation during endoscopic resection. Although traditional surgical resection offers a high rate of complete removal, it is often associated with greater trauma and insufficient functional preservation. With advances in endoscopic techniques, endoscopic submucosal excavation (ESE) has been increasingly used in the management of small rectal GISTs; however, successful preservation of the rectal serosal layer during ESE remains rarely reported.

**Case Presentation:**

A 51-year-old woman was found to have a 0.5 cm submucosal lesion located 12 cm from the anal verge during a screening colonoscopy. Endoscopic ultrasonography (EUS) revealed a heterogeneous hypoechoic lesion arising from the muscularis propria (4.7 × 4.0 mm). Contrast-enhanced MRI suggested a neuroendocrine tumor. After exclusion of contraindications, ESE was performed, achieving complete tumor removal with negative margins. Notably, the serosal layer of the rectal wall was fully preserved, preventing perforation. The mucosal defect was prophylactically closed with endoscopic clips. Postoperative recovery was uneventful, and no complications occurred.

**Conclusion:**

ESE can achieve complete resection of small rectal GISTs while preserving the serosal layer and rectal function. Although this case demonstrates the feasibility and safety of serosal-sparing ESE, further studies with larger sample sizes and long-term follow-up are warranted.

## Introduction

Gastrointestinal stromal tumor (GIST) is the most common mesenchymal neoplasm of the gastrointestinal tract, but rectal GISTs are uncommon, representing only about 5% of all cases. Most rectal GISTs are incidentally detected during colonoscopy (1). Based on tumor size, location, and mitotic index, GISTs are categorized into very low-, low-, intermediate-, and high-risk groups. Lesions smaller than 2 cm are generally defined as small GISTs, which are typically indolent or of low malignant potential, although some may exhibit local invasiveness (2). Surgical resection remains the standard treatment for GISTs, particularly for tumors larger than 2 cm. However, the optimal management of small rectal GISTs remains controversial. Some studies suggest that rectal GISTs have higher malignant potential and recurrence rates, recommending early intervention, while others advocate endoscopic resection for small, low-risk lesions (3). The management of rectal GISTs poses unique challenges. On one hand, their proximity to the anal canal and pelvic floor structures makes traditional surgery effective but potentially morbid, often leading to significant trauma or postoperative functional impairment (4). On the other hand, the rectal wall is relatively thin, and lesions commonly arise from the muscularis propria, increasing the risk of perforation during endoscopic procedures (5).

With the development of endoscopic techniques such as ESE and endoscopic full-thickness resection (EFTR), minimally invasive management of small rectal GISTs has become possible. However, intraoperative perforation has been reported in some cases, necessitating endoscopic closure or even surgical repair (6). Thus, achieving complete resection while minimizing perforation risk remains a critical concern. Here, we report a rare case of a small rectal GIST originating from the muscularis propria that was successfully resected by ESE with preservation of the serosal layer, thereby minimizing postoperative complications.

## Case description

**Figure 1:**
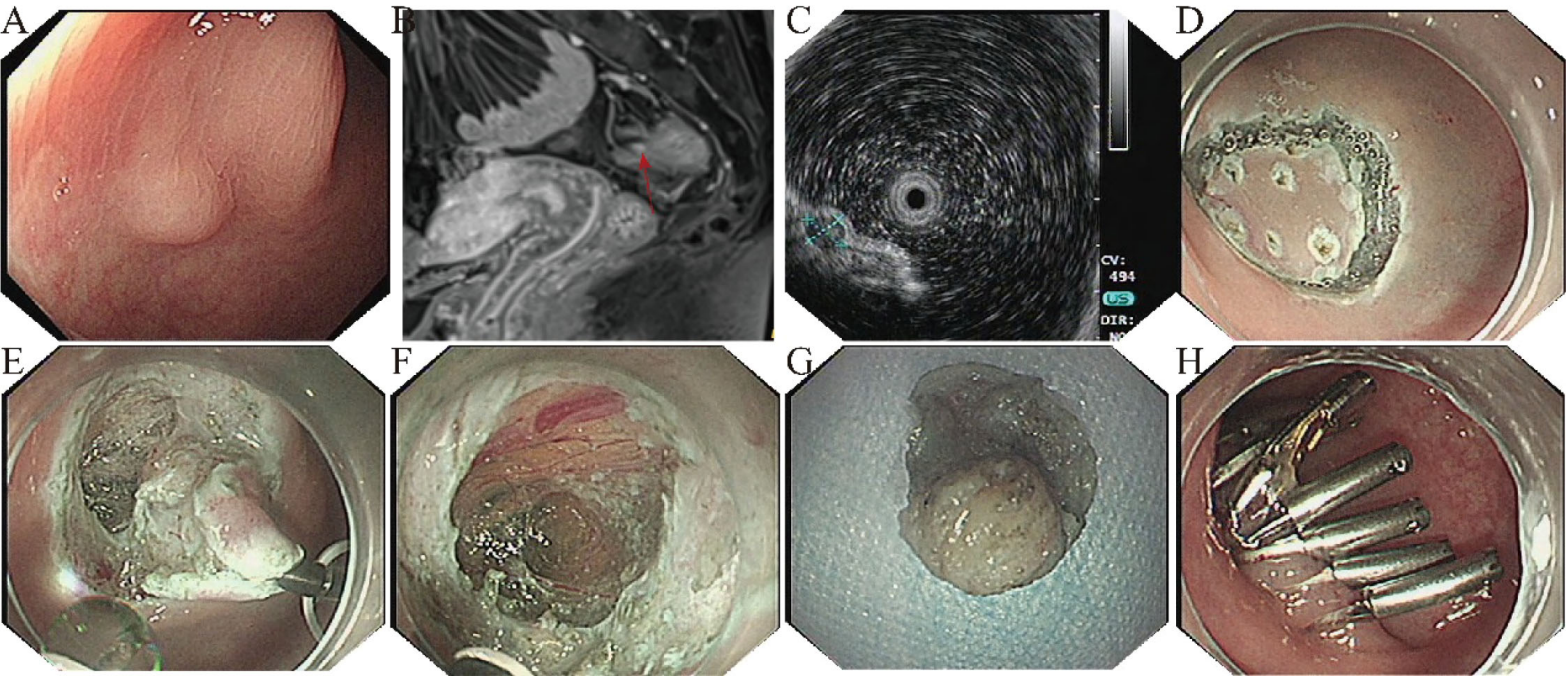
Endoscopic and imaging findings. **(A)** Under conventional white-light endoscopy, the lesion appeared as a submucosal bulge with a smooth surface; **(B)** Contrast-enhanced MRI showed a submucosal nodular lesion in the upper rectum (red arrow); **(C)** Endoscopic ultrasonography revealed a hypoechoic mass originating from the muscularis propria; **(D)** Intraoperative marking and mucosal incision; **(E)** Intraoperative dissection of the tumor; **(F)** After careful identification of the tumor boundary, the lesion was excised while preserving the transparent serosal layer of the rectal wall, with faint yellow peritoneal tissue visible externally; **(G)** Postoperative specimen of the tumor; **(H)** Closure of the wound with titanium clips.

A 51-year-old asymptomatic woman underwent a screening colonoscopy as part of a routine health check-up, which revealed a submucosal lesion approximately 12 cm from the anal verge. Under white light endoscopy, the lesion measured approximately 0.5 cm in diameter, with a smooth, pale surface (**Figure 1A**). Contrast-enhanced MRI revealed a small submucosal nodule in the upper rectum, showing high signal intensity on DWI with marked arterial-phase enhancement and decreased enhancement on the equilibrium phase, and the imaging features favored a neuroendocrine tumor (NET) in the differential diagnosis (**Figure 1B**). EUS demonstrated a hypoechoic lesion arising from the muscularis propria (4.7 × 4.0 mm) with heterogeneous internal echoes and a well-defined margin (**Figure 1C**). Laboratory tests, including complete blood count, coagulation profile, immunologic screening, and biochemical parameters, were within normal limits. Given the muscularis propria origin and the MRI differential favoring NET, together with the strong preference of the patient and her family for definitive diagnosis and treatment, endoscopic resection was chosen to obtain a histopathological diagnosis and achieve therapeutic intent.

After thorough discussion, the patient opted for endoscopic resection. The lesion was completely removed using ESE (**Figure 1E**). During the procedure, the rectal serosal layer was preserved intact (**Figure 1F**), and no perforation occurred, minimizing procedural trauma and postoperative risk. In brief, the procedure followed a “marking–submucosal injection–mucosal incision–stepwise dissection to expose the tumor–traction assistance–incision of the muscularis propria with dissection in the plane between the muscularis propria and the outer layer–hemostasis–central muscle-to-muscle approximation with a three-pronged clip–complete closure with standard through-the-scope clips” strategy; no pneumoperitoneum occurred.

**Figure 2:**
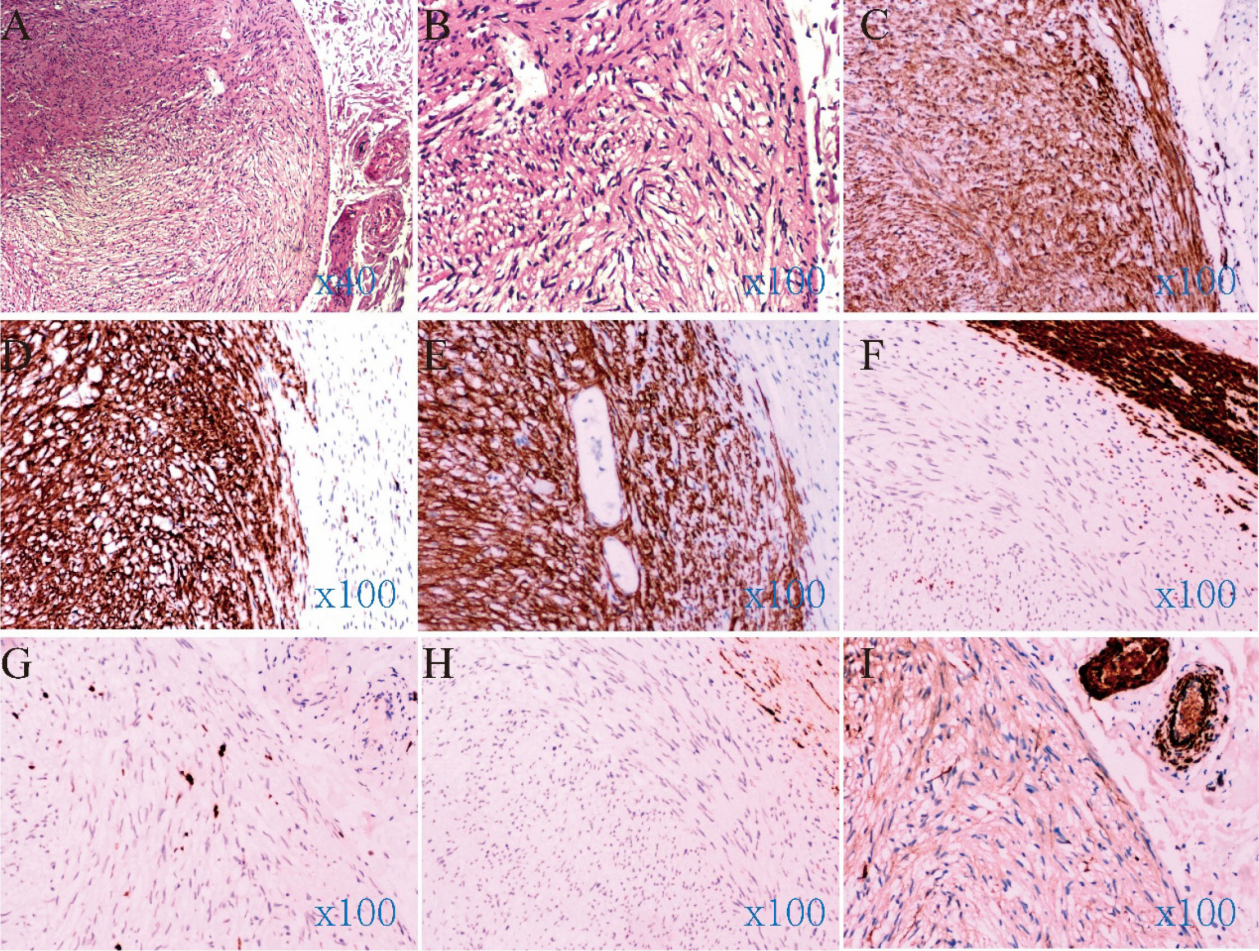
Histopathological and immunohistochemical findings. **(A)** Histopathological examination revealed a spindle cell tumor; **(B)** Under high-power microscopy, the nuclei appeared rod-shaped, and the cytoplasm was eosinophilic; **(C)** CD34 (+); **(D)** CD117 (+); **(E)** DOG-1 (+); **(F)** Desmin (-); **(G)** Ki-67 (-); **(H)** S-100 (-); **(I)** SMA (-).

Histopathological examination revealed a spindle cell tumor located predominantly within the submucosal tissue of the resected specimen (**Figures 2A, B**). Immunohistochemistry showed positivity for CD117, DOG1, and CD34, and negativity for S-100, Desmin, and SMA, with a Ki-67 index of approximately 1% (**Figures 2C–I**). The diagnosis was gastrointestinal stromal tumor of extremely low malignant potential (<5 mitoses/50 HPF). Postoperative recovery was uneventful. Supportive care was provided without antibiotics, and a liquid diet was resumed 48 hours after the procedure and then gradually advanced to a low-residue diet. The patient was discharged on postoperative day 7, and a telephone follow-up at 3 months indicated that she remained well without symptoms or related complications. She was advised to follow a soft, low-residue diet for 1 month, avoid heavy lifting and strenuous activity, and undergo surveillance colonoscopy at 6–12 months postoperatively.

## Discussion

This patient’s rectal lesion was incidentally discovered during colonoscopy and initially suspected to be a neuroendocrine tumor. EUS suggested a small (≈5 mm) hypoechoic lesion arising from the muscularis propria. Complete en bloc resection was achieved using ESE while preserving the serosal layer, effectively reducing the risk of postoperative perforation and related complications. Although sub-centimeter submucosal elevations can be managed with surveillance in selected cases, our patient had heterogeneous echogenicity on EUS with focal hyperechoic components, and contrast-enhanced MRI findings favored a neuroendocrine tumor in the differential diagnosis. In addition, after evaluations at multiple institutions, the patient and her family strongly requested definitive resection. Following shared decision-making and detailed counseling, we therefore proceeded with immediate endoscopic resection to establish a definitive diagnosis and achieve therapeutic intent.

GISTs most frequently occur in the stomach and small intestine, while rectal GISTs account for approximately 5% of all cases (7, 8). Most rectal GISTs are asymptomatic and are often discovered incidentally, particularly when smaller than 1 cm (9). Endoscopically, they are difficult to distinguish from leiomyomas and neuroendocrine tumors; thus, histopathological and immunohistochemical confirmation is required for definitive diagnosis (10). Histologically, rectal GISTs are typically composed of spindle cells with eosinophilic cytoplasm and elongated nuclei. Immunohistochemically, they are characterized by CD117 and DOG1 positivity (11, 12).

Surgical resection remains the mainstay of GIST management, with high rates of R0 resection (8, 13). However, given the complex pelvic anatomy, rectal surgery may cause significant trauma and functional impairment, including defecatory dysfunction. For small, low-risk lesions, traditional surgery may constitute overtreatment (14, 15). With advances in endoscopic technology, minimally invasive procedures such as ESE and EFTR have emerged as viable alternatives for selected cases (16, 17). Previous studies have demonstrated that ESE is a feasible therapeutic option for small rectal GISTs; however, perforation remains a major concern, particularly when the tumor originates from the muscularis propria, where the thin rectal wall increases the likelihood of full-thickness defects. Moreover, management of small rectal GISTs remains heterogeneous across guidelines and consensus statements: some favor a more proactive resection strategy, whereas others support EUS-based surveillance for small nodules, with intervention reserved for interval growth or high-risk features during follow-up. (15–17).

Nonetheless, previous reports have documented intraoperative perforation during ESE or EFTR, requiring endoscopic closure with clips or stents, or even surgical intervention (9, 18, 19). Although most patients recover uneventfully, these complications carry potential risks of peritoneal infection and functional damage. In the present case, careful dissection and precise delineation of tumor margins allowed for complete resection while maintaining the integrity of the serosal layer. The application of prophylactic clips was preventive rather than reparative, ensuring both R0 resection and reduced postoperative risk. Intraoperatively, a through-the-scope (TTS) twin clip was deployed centrally to achieve muscle-to-muscle apposition of the defect margins. The defect was then completely closed in a stepwise fashion using conventional endoscopic titanium clips.

Preservation of the serosal layer significantly reduces the likelihood of perforation and postoperative contamination while maintaining rectal structural integrity and physiological function (16). Nevertheless, this is a single case report. The feasibility of serosal preservation depends on multiple factors, including tumor size, location, operator experience, and endoscopic technique.

In summary, this case demonstrates that ESE can achieve complete resection of small rectal GISTs while preserving the serosal layer, thereby minimizing perforation risk and protecting rectal function. For centers with appropriate expertise and equipment, ESE may serve as a safe, effective, and minimally invasive therapeutic option for small rectal GISTs. Further studies with larger cohorts and long-term follow-up are warranted to validate its efficacy and safety (9, 20).

## Conclusion

We report a rare case of a small rectal GIST successfully treated by ESE with complete serosal layer preservation. This approach minimized postoperative complications and helped maintain rectal function, demonstrating the safety and feasibility of endoscopic management for GISTs in rare anatomical locations. The experience from this case may provide valuable reference for similar future cases.

## Data Availability

The original contributions presented in the study are included in the article/supplementary material. Further inquiries can be directed to the corresponding author.
